# *Gryllus bimaculatus*‐containing diets protect against dexamethasone‐induced muscle atrophy, but not high‐fat diet‐induced obesity

**DOI:** 10.1002/fsn3.3257

**Published:** 2023-02-04

**Authors:** Min Hee Kim, Su‐Jeong Kim, Si‐Hyun Kim, Woo‐Jae Park, Jung‐Soon Han

**Affiliations:** ^1^ Department of Biochemistry College of Medicine, Ewha Womans University Seoul South Korea; ^2^ Department of Biochemistry Chung‐Ang University College of Medicine Seoul South Korea; ^3^ Department of Human Ecology (Food Science and Nutrition) Korea University Seoul South Korea

**Keywords:** dexamethasone, edible insect, *Gryllus bimaculatus*, muscle atrophy, sarcopenia

## Abstract

Sarcopenia and obesity are emerging as major social problems. In this study, we examined whether *Gryllus bimaculatus* (GB), an edible insect, prevents dexamethasone‐induced muscle atrophy (sarcopenia) or high‐fat diet (HFD)‐induced obesity in mice. We generated a standard chow diet (SCD) + GB (85% SCD and 15% GB powder) and HFD + GB (85% HFD and 15% GB powder). SCD + GB feeding increased gains in body weight and white adipose tissue (WAT). Despite no difference in weight change between HFD + GB‐ and HFD‐fed mice, HFD + GB feeding aggravated insulin resistance compared with HFD feeding. SCD + GB or HFD + GB feeding did not change most gene expressions in the liver and WAT but did increase MyHC1 expression in the muscle, meaning that GB increased muscle generation. Therefore, we fed SCD + GB with dexamethasone, which induces muscle degeneration. As a result, muscle fiber size increased, as did grip strength compared with dexamethasone‐injected mice. In addition, SCD + GB reduced the expression of muscle degradation factors, such as atrogin1 and muscle RING‐finger protein 1 (MuRF1). Furthermore, SCD + GB feeding increased Akt, mTOR, and p70S6K phosphorylation and MyHC1 expression, meaning that it may have increased protein synthesis. In conclusion, GB has great potential for inhibiting dexamethasone‐induced muscle mass loss by increasing muscle protein synthesis and inhibiting muscle protein degradation.

## Graphical abstract

*Gryllus bimaculatus* containing diet prevented dexamethasone‐induced sarcopenia. It increased muscle synthesis and reduced muscle degradation.

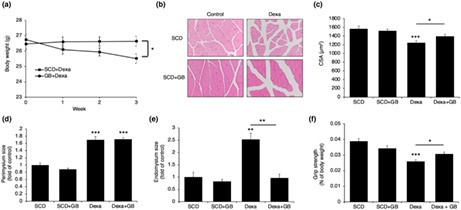

## 1 INTRODUCTION

Sarcopenia is the loss of muscle mass, quality, and strength (Morley et al., 2001). It is usually associated with aging, but there are multiple contributing conditions, including decreased physical activity, cellular senescence, increased cytokine activity (IL‐1β, IL‐6, and TNF‐α), decreased anabolic hormones (testosterone, growth hormone, and insulin growth factor), oxidative stress, mitochondrial dysfunction, fat accumulation, satellite cell dysfunction, and inadequate nutrition (Mankhong et al., 2020). The exact molecular mechanisms responsible for sarcopenia remain unknown but share some common trends with cancer‐associated cachexia (Peixoto da Silva et al., 2020), a complicated systemic disease involving various metabolic pathways (Peixoto da Silva et al., 2020) caused by anabolic signal resistance and negative energy balance. Skeletal muscle atrophy can be induced in vivo through the administration of dexamethasone, a glucocorticoid with potent immunosuppressant and anti‐inflammatory activities (Kaasik et al., 2007) that increases catabolic signals and decreases anabolic signals in muscle (Kaasik et al., 2007). So far, no effective therapeutic options have been developed to treat muscular wasting, and there are no FDA‐approved drugs or therapies in clinical practice.

*Gryllus bimaculatus* (GB), *Tenebrio molitor* (mealworm), *Alphitobius diaperinus*, and *Locusta migratoria* are examples of edible insects. These insects contain fat, vitamins, minerals, and large amounts of protein containing essential amino acids. Therefore, they can be used as functional foods. *T. molitor*‐derived protein supplementation and hydrolysate prevent muscle atrophy and increased myogenesis (Kim, Youngkyun, & Oh, 2020; Lee et al., 2021). However, the effects of other edible insects on muscle atrophy and myogenesis have not been examined. GB extract has been shown to provide protection from alcohol‐induced steatohepatitis (Hwang et al., 2019) and exerted a glucose‐lowering effect in streptozotocin‐induced diabetic mice (Park et al., 2020). It has also inhibited several inflammatory mechanisms in chronic arthritis models (Ahn et al., 2014), macrophage cell lines (Park & Han, 2021), colon epithelial cell lines (Kim et al., 2021), and human hepatoma cell lines (Kim et al., 2022). Because edible insects have proven to have various functions, edible insects are likely to be developed as functional foods and used as important resources in the future.

In this study, we examined the effects of GB‐containing diets on dexamethasone‐induced muscle atrophy and high‐fat diet (HFD)‐induced obesity to prove antisarcopenia and antiobesity effects as functional foods.

## 2 MATERIALS AND METHODS

### 2.1 Materials

Dexamethasone and anti‐α‐tubulin antibody (T9026) were purchased from Sigma‐Aldrich (St. Louis, MO). Anti‐phosphor‐Akt (Ser473) (4060), anti‐Akt (4691), anti‐phospho‐mTOR (Ser2448) (5536), anti‐mTOR (2983), anti‐phospho‐Smad2 (Ser465/467)/Smad3 (Ser423/425) (8828), anti‐Smad2/Smad3 (8685), anti‐phospho‐p70S6K (Thr389) (9205), and anti‐p70S6K (34475) antibodies were obtained from Cell Signaling Technology. Anti‐atrogin1 (sc‐166806), antimuscle RING‐finger protein 1 (MuRF1) (sc‐398608), and anti‐MyHC (sc‐376157) antibodies were obtained from Santa Cruz Biotechnology. Anti‐mouse‐horseradish peroxidase (HRP) (115–036‐003) and anti‐rabbit‐HRP (111–035‐003) antibodies were purchased from Jackson Laboratory (Bar Harbor, ME). GB was purchased from a cricket plantation. GB was used within 2 weeks after it became imago (adult cricket). After fasting for 2 days, GB was washed, steamed, and dried at 60°C. Afterward, they were pulverized with a blender (DA 5000; Daesung Artlon Co., Ltd.) and used in powder form.

### 2.2 Animal, diets, and dexamethasone injection

SCD + GB, composed of 15% GB powder and 85% standard chow diet (SCD; D12450K, Research Diet, Inc.), and HFD + GB, composed of 15% GB powder and 85% HFD (D12492, Research Diet, Inc.), were mixed and irradiated with gamma‐ray irradiation (Saeron Bio, Seongnam, South Korea). C57BL6 mice (male, 16−18 g, 6 weeks old) were purchased from Orient Bio (Seongnam), divided into four groups (each 10 mice), and allowed free access to SCD, SCD + GB, HFD, or HFD + GB. At the end of the 12th week, mice were anesthetized, and the liver, perigonadal adipose tissues, muscles (gastrocnemius), and sera were collected for further analyses. During diet feeding, metabolic studies were performed using the Labmaster system (TSE Systems GmbH). In sarcopenia experiments, 8‐week‐old C57BL6 mice were fed ab libitum either SCD or SCD + GB (each five mice) with intraperitoneal dexamethasone injection (10 mg/kg/week; Sun et al., 2014) for 3 weeks. Mice were anesthetized, and muscles (gastrocnemius) and sera were collected for further analyses. All animal experiments were approved according to the Animal Ethics Committee at Lee Gil Ya Cancer and Diabetes Institutes (LCDI‐2020‐0050).

### 2.3 Proximate analysis

The moisture content of each diet was measured after drying in an oven at 105°C. The crude ash was analyzed by the direct incineration method (JSMF‐140 T, JSR, Inc., Laboratory) at 600°C according to the American Association of Cereal Chemists (AACC) official methods (Boye et al., 2010). The crude protein was determined using a semi‐micro‐Kjeldahl method (Peralta et al., 2022) with a nitrogen factor of 6.25. The crude fat was determined using the Soxhlet extraction method (Peralta et al., 2022). Total carbohydrate was calculated by subtracting the sum of the moisture, ash, crude protein, and crude lipid content from the total weight of the food.

### 2.4 Glucose tolerance test (GTT)

The GTT was performed as previously described (Park et al., 2013). Briefly, mice were fasted for about 18 h, and then glucose (2.0 g/kg) was intraperitoneally injected. Serum glucose levels were measured using an automatic glucometer (Accu‐Chek Performa, Roche Diagnostics) at 0 (before glucose injection), 15, 30, 45, 60, 90, and 120 min.

### 2.5 Hematoxylin and eosin (H&E) staining

The liver, adipose tissues, and muscle (gastrocnemius) were fixed in 4% paraformaldehyde solution and embedded in paraffin blocks. Four‐micrometer‐thick sections were cut and stained with H&E. The cross‐sectional area (CSA), perimysium size, and endomysium size were measured using ImageJ software.

### 2.6 Triglyceride measurement

Triglyceride level in the liver was examined using a Triglyceride Quantification Colorimetric/Fluorometric Kit (BioVision).

### 2.7 Measurement of grip strength

To measure the muscle force, limb grip strength was determined using a grip strength meter (BIO‐G53, Bioseb) as previously described (Yeon et al., 2020).

### 2.8 Cell culture and dexamethasone treatment

The C2C12 myocyte cell line was purchased from the American Type Culture Collection (Manassas, VA) and maintained in Dulbecco's modified Eagle medium (Hyclone) with 10% fetal bovine serum and 1% penicillin/streptomycin solution (Hyclone). GB extract was prepared as previously described (Park & Han, 2021). Briefly, GB power was extracted using 70% ethanol at 4°C overnight. GB extract was diluted to a concentration of 200 mg/mL using 1X PBS (1:2 ~ 1:10) and filtered through the 0.45 μm syringe filter. GB extract (200 μg/mL) and dexamethasone (1 μM) were co‐treated for 12 h.

### 2.9 Western blotting

Liver, perigonadal adipose tissues, muscles (gastrocnemius), and C2C12 cells were lysed using RIPA buffer (50 mM Tris‐Cl, pH 7.5; 150 mM NaCl, 1% Nonidet P‐40, 0.5% sodium deoxycholate, and 0.1% SDS) containing protease and phosphatase inhibitors (Sigma‐Aldrich). After centrifugation (10,000 ***g***, 4°C, 10 min), proteins (50 μg) were loaded and electrophoresed on 10% SDS polyacrylamide gels and then transferred to nitrocellulose (NC) membranes (Bio‐Rad Laboratories). Membranes were blocked with 5% bovine serum albumin (Sigma‐Aldrich) in TBST (TBS with 0.1% Tween 20) for 1 h at room temperature and further incubated with primary antibodies (1:1000 dilutions) at 4°C overnight. Incubation with secondary antibodies occurred at room temperature for 1 h. Protein bands were detected using the EzWestLumi Plus Reagents (ATTO Corp.) on the ChemiDoc MP imaging system (Bio‐Rad Laboratories).

### 2.10 Real‐time PCR (qPCR)

Total mRNA was extracted from the liver, perigonadal adipose tissues, and muscles using the RNeasy Kit, RNeasy Lipid Kit, and RNeasy Fibrous Kit (Qiagen), respectively. cDNA was synthesized using the ReverTra Ace qPCR RT Master Mix (Toyobo). qPCR was performed using the Thunderbird SYBR qPCR Mix (Toyobo) in a Bio‐Rad CFX96 System (Bio‐Rad Laboratories). Thermal cycling conditions consisted of a 1‐min hold at 95°C, followed by 40 cycles of 15 s at 95°C, and 45 s at 60°C. Relative gene expression was calculated using the 2^−ΔΔ*C*t^ method (Livak & Schmittgen, 2001). The gene expression in hepatic and adipose tissues was normalized to *GAPDH*, but that in muscle was normalized to the *PPIB* (cyclophilin B gene). The primers used in this study are described in Table 1.

**TABLE 1:** Primers used in this study for real‐time PCR.

Gene	Primer sequences	Accession number	References
*SREBP‐1a*	F: 5′‐TAGTCCGAAGCCGGGTGGGCGCCGGCGCCAT‐3′ R: 5′‐GATGTCGTTCAAAACCGCTGTGTGTCCAGTTC‐3′	NM_011480	Shimomura et al. (1997)
*SREBP‐1c*	F: 5′‐ATCGGCGCGGAAGCTGTCGGGGTAGCGTC‐3′ R: 5′‐ACTGTCTTGGTTGTTGATGAGCTGGAGCAT‐3′	NM_011480	Shimomura et al. (1997)
*FAS*	F: 5′‐GATGACACCAGCTTTGCCAA‐3′ R: 5′‐CAGTGAGTTGAGGACCAGGT‐3′	NM_007988	
*SCD‐1*	F: 5′‐GCGATACACTCTGGTGCTCA‐3′ R: 5′‐TATTCTCCCGGGATTGAATG‐3′	NM_009127	
*PPARα*	F: 5′‐CATGAACAAGGTCAAGGCCC‐3′ R: 5′‐TTCTCGGCCATACACAAGGT‐3′	NM_011144	
*PPAR𝛾*	F: 5′‐CCATTCACAAGAGCTGACCC‐3′ R: 5′‐AAGGTGGAGATGCAGGTTCT‐3′	NM_011146	
*CD36*	F: 5′‐TGCTCTCCCTTGATTCTGCT‐3′ R: 5′‐CTCCAAACACAGCCAGGACT‐3′	NM_007643	
*FATP5* *(slc27a5)*	F: 5′‐AAAGCTGAAGGATGCCGTAA‐3′ R: 5′‐CCAACCCCAGAAACACACTC‐3′	NM_009512	
*Acox1*	F: 5′‐TTTTCTTGAGACAGGGCCCA‐3′ R: 5′‐GTTCCAACTAGCCAGGCATG‐3′	NM_015729	
*Acox2*	F: 5′‐CAGAAGCCTCTCCCTCAGTC‐3′ R: 5′‐TATAGACTTCTGGGCAGCGG‐3′	NM_053115	
*TNF‐α*	F: 5′‐GCAAGCTTCGCTCTTCTGTCTACTGAACTT‐3′ R: 5′‐GCTCTAGAATGAGATAGCAAATCGGCTGAC‐3′	NM_013693	Hazlett et al. (2010)
*IL‐1β*	F: 5′‐CGGACCCCAAAAGATGAAGG‐3′ R: 5′‐GCTCTTGTTGATGTGCTGCT‐3′	NM_008361	
*IL‐6*	F: 5′‐GGAGCCCACCAAGAACGATA‐3′ R: 5′‐TTCTTGGGACTGATGCTGGT‐3′	NM_031168	
*ATGL*	F: 5′‐CCATCTGCCTTCCAGACTGT‐3′ R: 5′‐TGGGTAGGGCCTCACTGTAG‐3′	NM_025802	
*HSL*	F: 5′‐CTTCGGGGAGCACTACAAAC‐3′ R: 5′‐CCACGCAACTCTGGGTCTAT‐3′	NM_010719	
*PLIN1* (Perilipin)	F: 5′‐CCAGGCTGTCTCCTCTACCA‐3′ R: 5′‐GCGGCACATAGTGTACCACA‐3′	NM_175640	
*MuRF1* *(Trim63)*	F: 5′‐AGGACTCCTGCAGAGTGACCAA‐3′ R: 5′‐TTCTCGTCCAGGATGGCGTA‐3′	NM_001039048	Yeon et al. (2020)
*MAFbx* (Atrogin)	F: 5′‐GCAAACACTGCCACATTCTCTC‐3′ R: 5′‐CTTGAGGGGAAAGTGAGACG‐3′	NM_026346	Yeon et al. (2020)
*MSTN* (Myostatin)	F: 5′‐GGCCATGATCTTGCTGTAA‐3′ R: 5′‐TTGGGTGCGATAATCCAGTC‐3′	NM_010834	Yeon et al. (2020)
*MyHC1* *(Myh7)*	F: 5′‐CCAAGGGCCTGAATGAGGAG‐3′ R: 5′‐GCAAAGGCTCCAGGTCTGAG‐3′	NM_080728	Yeon et al. (2020)
*MyHC2A* *(Myh2)*	F: 5′‐AAGCGAAGAGTAAGGCTGTC‐3′ R: 5′‐GTGATTGCTTGCAAAGGAAC‐3′	NM_001039545	Yeon et al. (2020)
*MyHC2B* *(Myh4)*	F: 5′‐ACAAGCTGCGGGTGAAGAGC‐3′ R: 5′‐CAGGACAGTGACAAAGAACG‐3′	NM_010855	Yeon et al. (2020)
*PPIB* (Cyclophilin B)	F: 5′‐TGGAGAGCACCAAGACAGACA‐3′ R: 5′‐TGCCGGAGTCGACAATGAT‐3′	NM_011149	Yeon et al. (2020)
*GAPDH*	F: 5′‐ACTCACGGCAAATTCAACGG‐3′ R: 5′‐ATGTTAGTGGGGTCTCGCTC‐3′	NM_001289726	

*Note*: F, forward; R, reverse.

### 2.11 Statistical analyses

All experiments were repeated independently in triplicates, and the data were expressed as mean ± standard error of the mean (SEM). Statistical significance was calculated using analysis of variance (ANOVA), followed by Tukey's post hoc test, or two‐way ANOVA (GraphPad Prism 6.0; GraphPad Software). A *p* < .05 indicated statistically significant.

## 3 RESULTS

### 3.1 HFD + GB feeding did not affect obesity, but aggravated insulin resistance

**FIGURE 1:**
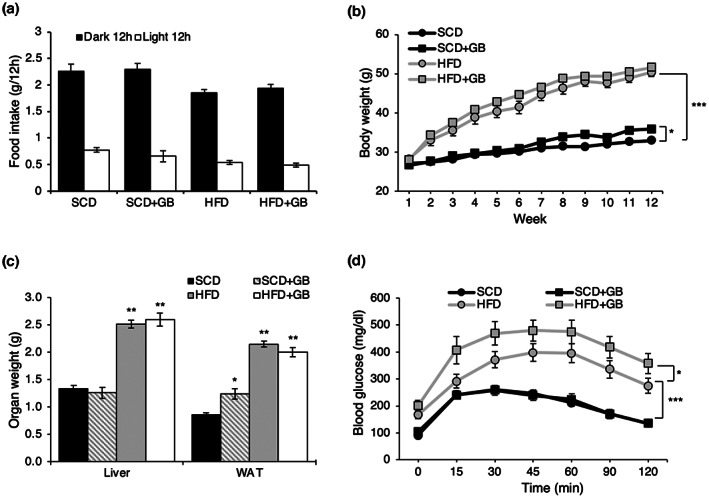
Effect of SCD + GB and HFD + GB on obesity. After SCD, SCD + GB (85% SCD, 15% GB powder), HFD, HFD + GB (85% HFD, 15% GB powder) feeding for 12 weeks, (a) food intake, (b) body weight changes, (c) liver, perigonadal WAT weights, and (d) glucose tolerance test (GTT) were examined in mice (*n* = 5, *n* = 10, *n* = 4, *n* = 10, respectively). Values expressed as mean ± SEM; *p < .05, **p < .01. All experiments were performed in triplicate. GB, *Gryllus bimaculatus*; HFD, high‐fat diet; SCD, standard chow diet; WAT, white adipose tissue.

To examine whether the GB extract affected HFD‐induced obesity and fatty liver formation, mice were administered SCD + GB and HFD + GB for 12 weeks. The contents of the prepared diets were analyzed by proximate analysis. Compared to SCD, SCD + GB contains more protein, fat, and less carbohydrate with similar ash content. HFD + GB contains more moisture, protein, and less fat compared to HFD (Table 2). The amount of food intake was evaluated during the experiment. Food intake in SCD + GB‐ and HFD + GB‐fed mice was not different from that in SCD‐ and HFD‐fed mice, respectively (Figure 1a). At the end of the 12th week, more body weight gain was shown in the SCD + GB‐fed mice compared to SCD‐fed mice (Figure 1b). However, there were no differences in the body weight gain between the HFD‐fed mice and the HFD + GB‐fed mice (Figure 1b). Interestingly, SCD + GB increased the weight of white adipose tissue (WAT) only, and there were no differences in organ weight between HFD + GB‐ and HFD‐fed mice (Figure 1c). However, HFD + GB‐fed mice showed higher insulin resistance in GTT (Figure 1d).

**TABLE 2:** Proximate composition of SCD, SCD + GB, HFD, and HFD + GB.

Sample	Properties (%)				
	Moisture	Crude ash	Crude protein	Crude fat	Carbohydrate
SCD	7.85 ± 0.12	5.88 ± 0.11	20.30 ± 1.09	2.77 ± 0.98	63.18 ± 0.98
SCD + GB	5.04 ± 3.02	5.59 ± 0.21	22.75 ± 1.68	6.41 ± 0.58	60.22 ± 2.48
HFD	7.80 ± 1.07	3.64 ± 0.38	22.30 ± 1.66	30.58 ± 0.20	35.69 ± 2.43
HFD + GB	12.70 ± 0.72	4.12 ± 0.29	24.09 ± 0.33	24.35 ± 1.10	34.75 ± 1.40

Abbreviations: GB, *Gryllus bimaculatus*; HFD, high‐fat diet; SCD, standard chow diet.

### 3.2 SCD + GB did not affect fat metabolism in liver and adipose tissues, but increased myosin heavy chain 1 (MyHC1) in muscle

**FIGURE 2:**
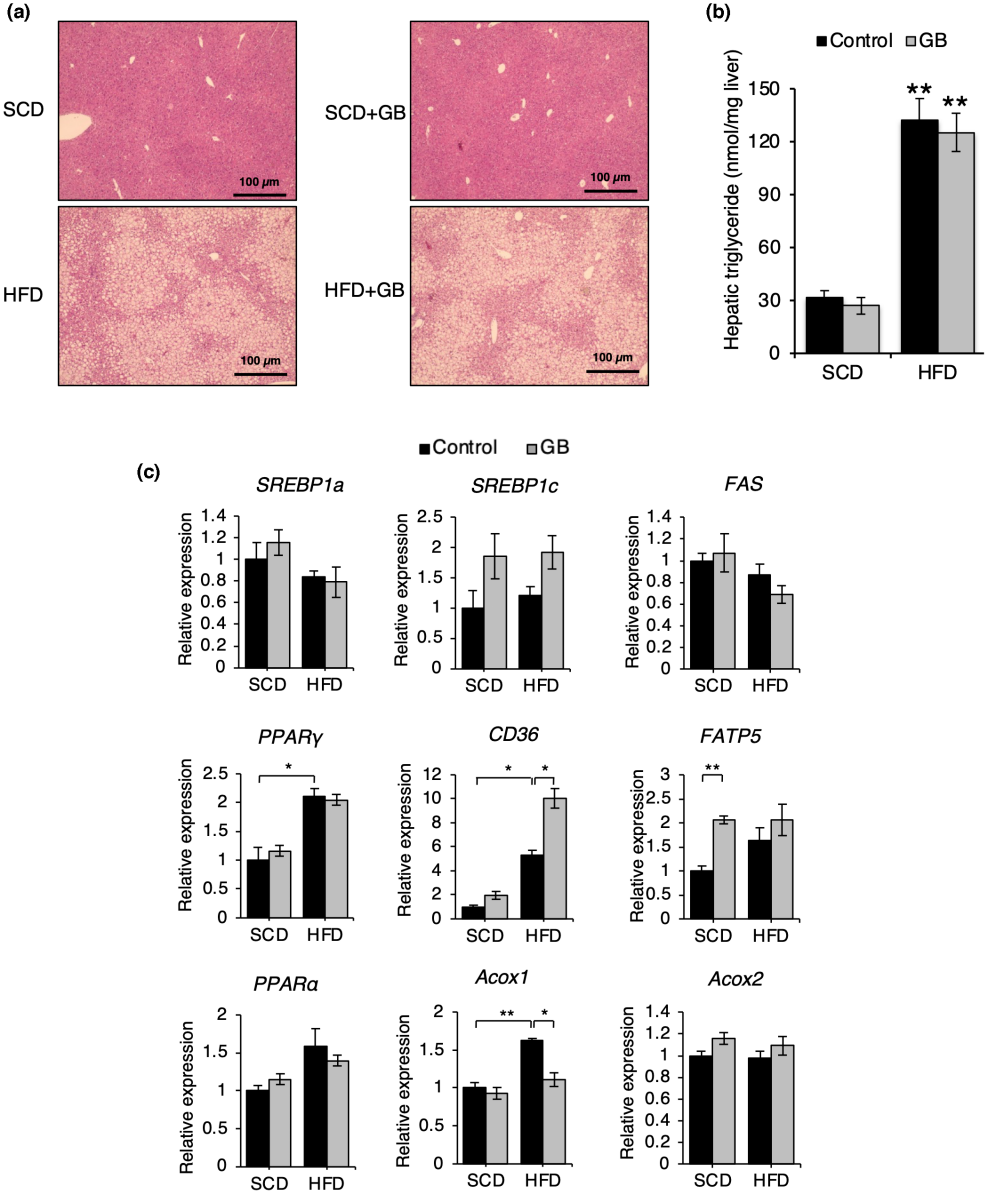
Effect of SCD + GB and HFD + GB on fatty liver formation. After SCD + GB and HFD + GB feeding for 12 weeks, histological analysis (scale bar, 100 μm) (a) and triglyceride amount (b) of liver were examined. (c) Relative mRNA levels of the genes related to lipogenesis (*SREBP‐1a*, *−1c*, *FAS*, *PPARγ*), fatty acid uptake (*CD36*, *FATP5*), and fatty acid oxidation (*Acox1*, *Acox2*, *PPARα*) in the livers of mice (*n* = 3). Values expressed as mean ± SEM; *p < .05, **p < .01. All experiments were performed in triplicates. Acox, acyl‐CoA oxidase; CD36, cluster of differentiation 36; FAS, fatty acid synthase; FATP, fatty acid transport protein; GB, *Gryllus bimaculatus*; HFD, high‐fat diet; PPAR, peroxisome proliferator‐activated receptor; SCD, standard chow diet; SREBP, sterol regulatory element‐binding protein.

**FIGURE 3:**
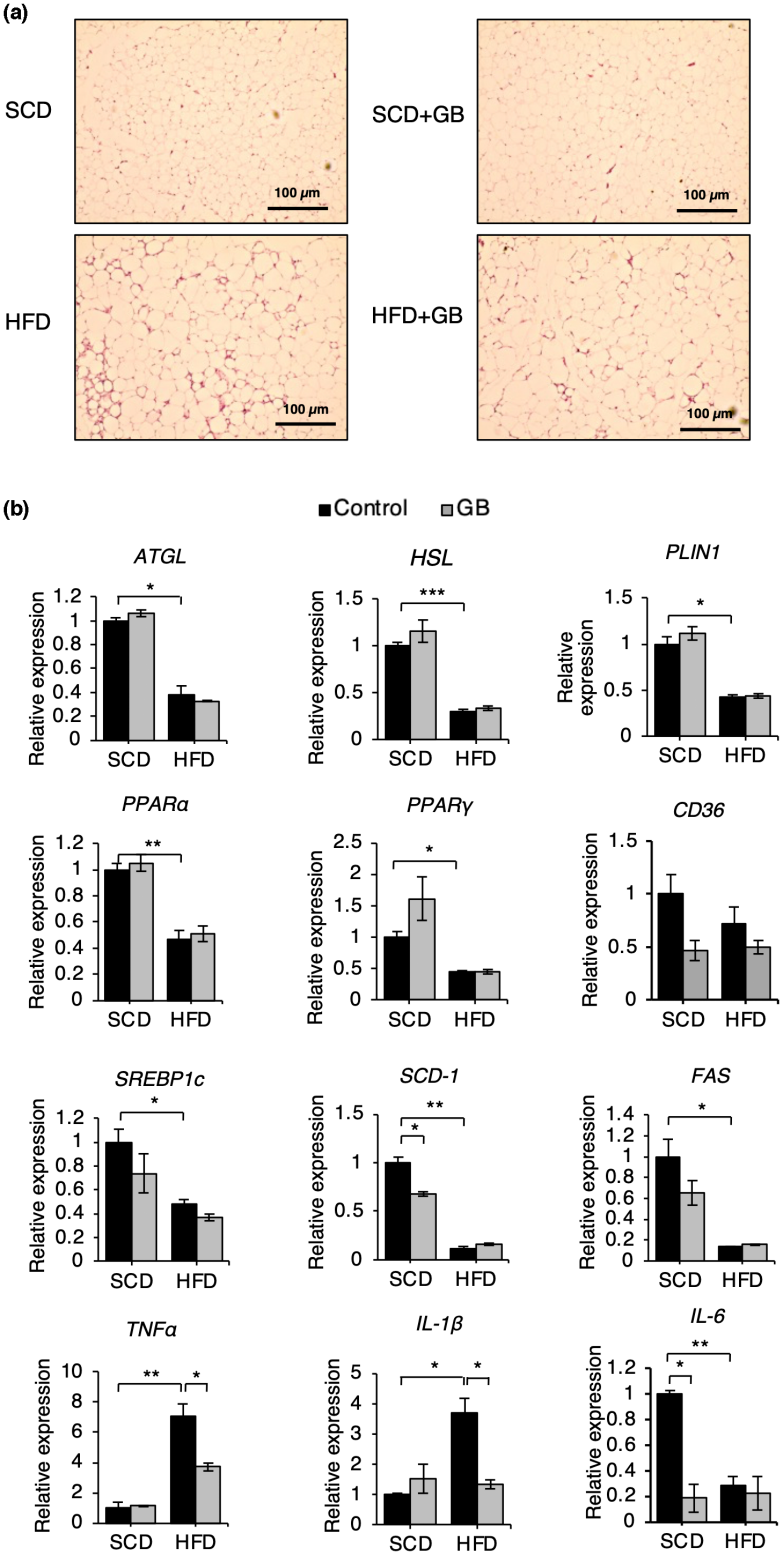
Effect of SCD + GB and HFD + GB on adipose tissue weight. (a) Histologic section of perigonadal WAT (scale bar, 100 μm). (b) Relative mRNA levels of the genes related to lipolysis (*HSL*, *Atgl*), lipogenesis (*SREBP‐1c*, *FAS*, *SCD‐1*, *PPARγ*, *PLIN1*), fatty acid uptake (*CD36*), fatty acid oxidation (*PPARα*), and inflammation (*TNFα*, *IL‐1β*, *IL‐6*) in perigonadal WAT of mice with SCD + GB and HFD + GB feeding (*n* = 3). Values expressed as mean ± SEM; *p < .05, ** *p* < .01, ***p < .001. All experiments were performed in triplicate. ATGL, adipose triglyceride lipase; CD36, cluster of differentiation 36; FAS, fatty acid synthase; GB, *Gryllus bimaculatus*; HFD, high‐fat diet; HSL, hormone‐sensitive lipase; IL, interleukin; PPAR, peroxisome proliferator‐activated receptor; SCD, standard chow diet; SCD‐1, stearoyl‐CoA desaturase 1; TNF, tumor necrosis factor; WAT, white adipose tissue.

**FIGURE 4:**
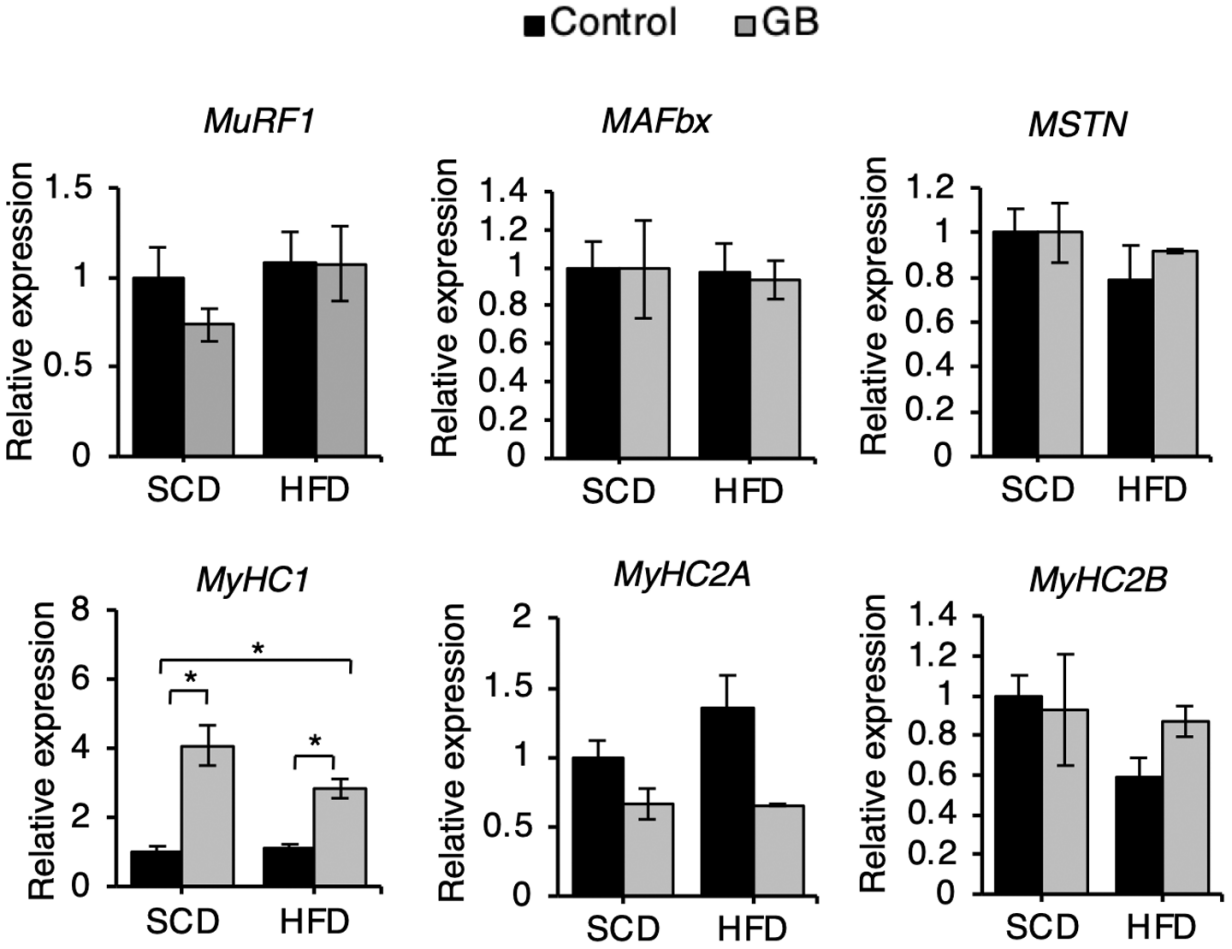
Effect of SCD + GB and HFD + GB on MyHC1 expression. Relative mRNA levels of the genes related to muscle degradation factors (*MuRF1*, *MAFbx* (atrogin1), *MSTN* (myostatin)) and myosin heavy chain (*MyHC1*, *MyHC2A*, *MyHC2B*) in the muscle of mice with SCD + GB and HFD + GB feeding (*n* = 3). Values expressed as mean ± SEM; *p < .05. All experiments were performed in triplicate. Abbreviations: GB, *Gryllus bimaculatus*; HFD, high‐fat diet; MuRF1, muscle RING‐finger protein‐1; MyHC, myosin heavy chain; SCD, standard chow diet.

SCD + GB and HFD + GB did not affect fatty liver formation (Figure 2a) and triglyceride accumulation (Figure 2b). SCD + GB increased fatty acid transport protein 5 (*FATP5*) expression, and HFD + GB increased *CD36* expression. HFD + GB reduced acyl‐CoA oxidase 1 (*Acox1*) expression. However, both diets did not affect most of the gene expressions of fatty acid metabolism (*SREBP‐1a*, *1c*, *FAS*, *PPARγ*, *PPARα*, *Acox2*) (Figure 2c). SCD + GB did not affect the size of WAT (Figure 3a) and the gene expressions of fatty acid metabolism (*SREBP‐1c*, *FAS*, *PPARγ*, *PPARα*, *CD36*, *PLIN1*(perilipin)) (Figure 3b). HFD + GB reduced *TNFα* and *IL‐1β* expressions compared to HFD in WAT (Figure 3b), meaning that HFD + GB can reduce inflammation. In the light that SCD + GB and HFD + GB did not affect fat accumulation in the liver and WAT, we examined the gene expression patterns pertaining to muscle degradation (*MuRF1*, *atrogin1*, *myostatin*) and muscle generation (*MyHC1*, *MyHC2A*, *MyHC2B*). Both SCD + GB and HFD + GB increased *MyHC1* expression (Figure 4).

### 3.3 SCD + GB prevented dexamethasone‐induced muscle atrophy

**FIGURE 5:**
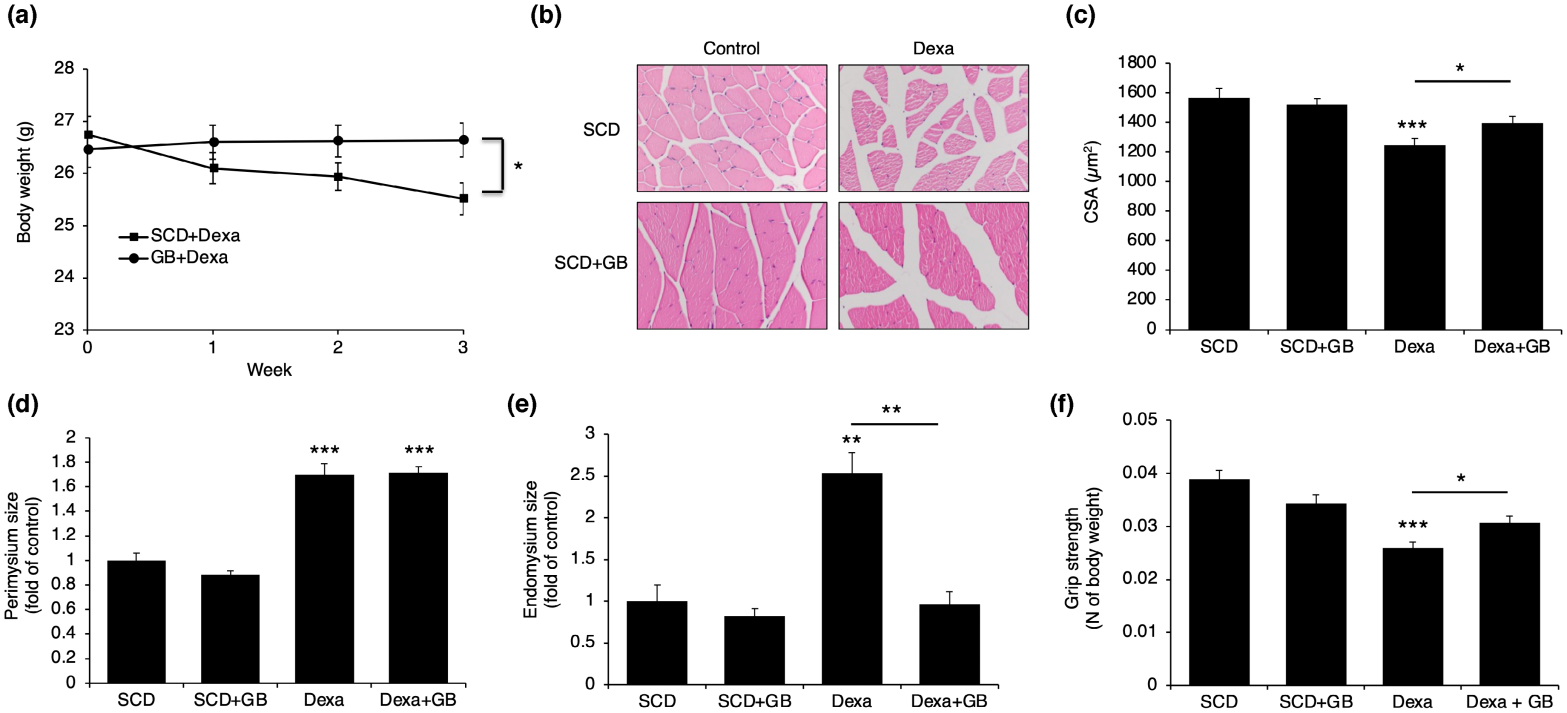
SCD + GB prevents Dexa‐induced muscle atrophy. After SCD + GB feeding with Dexa injection, (a) body weight changes were monitored weekly (*n* = 5). (b) Histologic section of gastrocnemius was shown. CSA (c), perimysium size (d), and endomysium size (e) were measured using ImageJ program (*n* = 4). (f) Forelimb grip strength tests were examined (*n* = 4). Values expressed as mean ± SEM; *p < .05, **p < .01, ***p < .001. All experiments were performed in triplicate. CSA, cross‐sectional area; Dexa, dexamethasone; GB, *Gryllus bimaculatus*; SCD, standard chow diet.

Having shown that SCD + GB increased MyHC1, we fed either SCD or SCD + GB to the mice with dexamethasone injection. Dexamethasone injection reduced body weight, but SCD + GB prevented dexamethasone‐induced body weight loss (Figure 5a). Myocyte size decreased after dexamethasone injection, but SCD + GB prevented dexamethasone‐induced muscle atrophy (Figure 5b,c). SCD + GB recovered both the size of the endomysium (Figure 5e) and the grip strength (Figure 5f). However, SCD + GB did not affect the size of the perimysium (Figure 5d).

### 3.4 SCD + GB reduced muscle degradation but increased muscle synthesis

**FIGURE 6:**
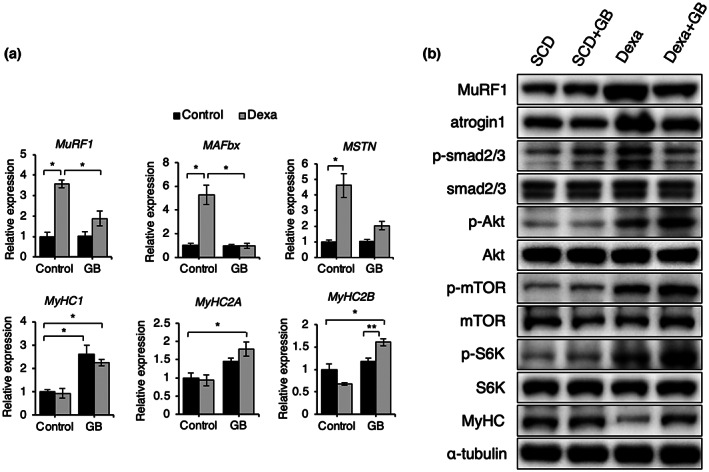
SCD + GB reduces Dexa‐induced protein degradation and increases protein synthesis. (a) Relative mRNA levels of the genes related to muscle degradation factors (*MuRF1*, *MAFbx* (atrogin1), *MSTN* (myostatin)), and myosin heavy chain (*MyHC1*, *MyHC2A*, *MyHC2B*) in the muscle of mice fed SCD + GB with Dexa injection (*n* = 3). (b) Representative western blots of the indicated proteins after SCD + GB feeding with Dexa injection. Values expressed as mean ± SEM; *p < .05, **p < .01. All experiments were performed in triplicate. Dexa, dexamethasone; GB, *Gryllus bimaculatus*; mTOR, mammalian target of rapamycin; MuRF1, muscle RING‐finger protein‐1; MyHC, myosin heavy chain; SCD, standard chow diet.

**FIGURE 7:**
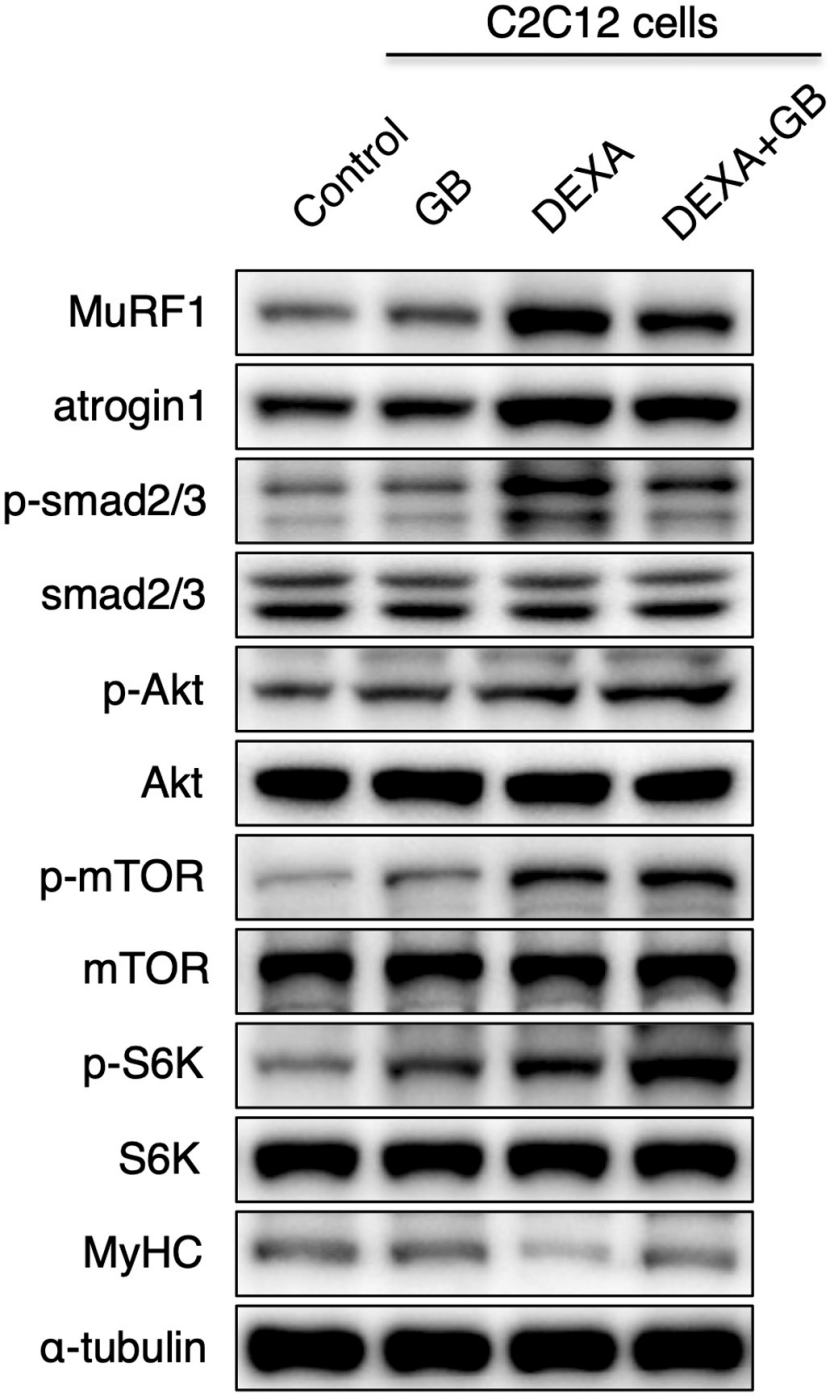
GB extract reduces Dexa‐induced protein degradation but increases protein synthesis. Representative western blots of the indicated proteins after 200 μg/mL GB extract treatment with 1 μM Dexa. Values expressed as mean ± SEM; *p < .05, **p < .01. All experiments were performed in triplicate. Dexa, dexamethasone; GB, *Gryllus bimaculatus*; mTOR, mammalian target of rapamycin; MuRF1, muscle RING‐finger protein‐1; MyHC, myosin heavy chain.

Muscle is maintained by protein synthesis and degradation. Myostatin, atrogin1, and MuRF1, muscle degradation factors (Malavaki et al., 2015), can be induced by dexamethasone treatment. The Akt and the mammalian target of rapamycin (mTOR) pathways play an important role in muscle protein synthesis by increasing MyHC expressions (Malavaki et al., 2015). The transforming growth factor β (TGF‐β) signaling pathway induced the phosphorylation of Smad2/3, which further increased MuRF1 and atrogin1 expressions (Hoogaars & Jaspers, 2018). Therefore, we examined the expressions of MuRF1 and atrogin1 and the phosphorylation of Smad2/3, Akt, mTOR, and S6K in muscle. Dexamethasone injection increased MuRF1, atrogin1, and myostatin levels, but SCD + GB reduced these levels (Figure 6a,b). Moreover, dexamethasone increased the phosphorylation of Smad2/3, but SCD + GB reduced the phosphorylation of Smad2/3. In the protein synthesis pathways, the MyHC1 level was elevated in the SCD + GB‐fed group, and the phosphorylation of Akt, mTOR, and S6K was also increased (Figure 6a,b). SCD + GB recovered the MyHC protein level after dexamethasone injection (Figure 6b). These signaling pathways were re‐examined in the C2C12 cell line with dexamethasone treatment. C2C12 cells treated with GB extract showed increased muscle generation based on the elevated Akt/mTOR/S6K pathway and MyHC level. Moreover, GB extract reduced muscle degradation, as seen by the reduced MuRF1 and atrogin1 expressions, and decreased Smad2/3 phosphorylation level (Figure 7).

## 4 DISCUSSION

Sarcopenia is a muscle disorder characterized by progressive and generalized loss of skeletal muscle mass, strength, and quality. Primary sarcopenia (aging associated) is generally mild but may accelerate due to genetic, lifestyle, and environmental factors. Secondary sarcopenia describes muscle wasting (pathophysiological muscle loss).

In this study, we showed that the diets containing 15% GB (SCD + GB and HFD + GB) prevented dexamethasone‐induced sarcopenia but not HFD‐induced obesity. Mice fed with SCD + GB for 12 weeks presented increased body weight and WAT weight. Furthermore, SCD + GB increased MyHC1 expression in skeletal muscle. However, no specific pathways in the liver and adipose tissues were changed between the HFD‐ and HFD + GB‐fed groups. Therefore, GB‐containing diets have great potential to increase muscle mass. Under normal conditions, the nutrition from GB powder can be used for triglyceride synthesis in WAT, whereas in sarcopenia, it can be used for muscle synthesis by activating the Akt/mTOR/S6K signaling pathway, which is upstream of MyHC (Liu et al., 2021). SCD + GB also reduced muscle degradation by reducing muscle degradation factors (MuRF1, atrogin1). Thus, the GB‐containing diets helped prevent dexamethasone‐induced sarcopenia by affecting both muscle synthesis and degradation.

GB powder has a high protein content with many essential amino acids, including valine, isoleucine, and leucine (Kim, Kim, & Han, 2020). These branched‐chain amino acids are oxidized in muscle and used as an energy source. GB powder also contains high amounts of oleic acid and linoleic acid (Park & Han, 2021). Oleic acid increases MyHC1 expression (Watanabe et al., 2020) and, along with linoleic acid, prevents muscle atrophy by preventing reactive oxygen species generation (Lee et al., 2017, 2022). Therefore, some nutritional factors of GB extract, such as high protein and unsaturated fatty acids, might affect muscle protein synthesis and muscle degradation.

Larvae of the edible insect *T. molitor* prevented HFD‐induced obesity through AMP‐activated protein kinase and mitogen‐activated protein kinases (Seo, Goo, et al., 2017), and the intracerebroventricular administration of *T. molitor* larvae extract suppresses appetite by reducing neuropeptide Y (Seo, Kim, et al., 2017). Moreover, *Allomyrina dichotoma* larvae reduce HFD‐induced body weight gain (Yoon et al., 2015) and regulate the antagonizing effects of ghrelin‐induced feeding behavior (J. Kim et al., 2016). In contrast to these outcomes of consuming *T. molitor* and *A. dichotoma*, mice ate the same amounts of GB‐containing diets and control diets (SCD or HFD), meaning that GB‐containing diets did not affect appetite or feeding behavior. Therefore, whether these insects share the same antiobesity mechanisms remains to be studied. The differences in the biological effects among these insects may be due to differences in their bioactive compounds.

Expression of SREBP‐1 and PPARs was unchanged in the liver and WAT with HFD and HFD + GB feeding, meaning that GB‐containing diets did not affect fat‐related transcriptional factors. Similarly, SCD + GB did not affect genes related to fatty acid oxidation. However, GB‐containing diets inhibited the expressions of inflammation‐related genes, such as TNF‐α and IL‐1β, in adipose tissues. Therefore, GB did not have antiobesity effects, but it had anti‐inflammatory effects as previously described (Park & Han, 2021). Other edible insects have also been shown to inhibit inflammation. For example, *T. molitor* and *A. dichotoma* reduce both HFD‐induced steatohepatitis and hepatic inflammation (Lee et al., 2020). GB also protects against alcohol‐induced liver damage (Hwang et al., 2019) and chronic arthritis (Ahn et al., 2014). Therefore, edible insects contain valuable bioactive compounds that inhibit inflammation.

In conclusion, GB may help increase muscle synthesis by activating the Akt/mTOR/S6K pathway and prevent the activation of muscle degradation factors (MuRF1, atrogin1) by dexamethasone. Thus, our data suggest that GB can be used as a functional food for muscle atrophy or sarcopenia, but further research on humans is needed for clinical application. Clinical trials are, therefore, required to confirm the effectiveness of GB for patients with sarcopenia and muscle atrophy.

## CONFLICT OF INTEREST

The authors declare that they do not have any conflict of interest.

## ETHICS STATEMENT

All animal experiments were reviewed and approved by the Animal Ethics Committee at Lee Gil Ya Cancer and Diabetes Institutes (LCDI‐2020‐0050).

## Data Availability

Data are openly available in a public repository that issues datasets with DOIs.
